# Conditional analysis of the major histocompatibility complex in rheumatoid arthritis

**DOI:** 10.1186/1753-6561-3-s7-s36

**Published:** 2009-12-15

**Authors:** Kimberly E Taylor, Lindsey A Criswell

**Affiliations:** 1 The Rosalind Russell Medical Research Center for Arthritis, University of California San Francisco, 374 Parnassus Ave, San Francisco, California 94143 USA

## Abstract

We performed a whole-genome association study of rheumatoid arthritis susceptibility using Illumina 550k single-nucleotide polymorphism (SNP) genotypes of 868 cases and 1194 controls from the North American Rheumatoid Arthritis Consortium (NARAC). Structured association analysis with adjustment for potential population stratification yielded 200 SNPs with *p *< 1 × 10^-8 ^for association with RA, all of which were on chromosome 6 in a 2.7-Mb region of the major histocompatibility complex (MHC). Given the extensive linkage equilibrium in the region and known risk of *HLA-DRB1 *alleles, we then applied conditional analyses to ascertain independent signals for RA susceptibility among these 200 candidate SNPs. Conditional analyses incorporating risk categories of the *HLA-DRB1 *"shared epitope" revealed three SNPs having independent associations with RA (conditional *p *< 0.001). This supports the presence of significant effects on RA susceptibility in the MHC in addition to the shared epitope.

## Background

Rheumatoid arthritis (RA) is a chronic systemic autoimmune disease characterized by damage to synovial joints as well as extraarticular manifestations. The strongest known genetic risk factor is the *HLA-DRB1 *gene on chromosome 6, namely a set of alleles sharing a common sequence known as the shared epitope (SE) [1]. Recent whole-genome association studies have revealed new risk genes outside of the HLA region [2-4], and some studies have also provided evidence of additional influences from the HLA class III and class I regions [5,6]. In this analysis we sought first to identify and/or validate RA risk alleles throughout the genome, and then to identify independent associations with RA susceptibility in the major histocompatibility complex (MHC) in addition to the SE.

## Methods

Illumina 550k genotyping data from a whole-genome association study by North American Rheumatoid Arthritis Consortium (NARAC) [3] was used for this study as part of the Genetic Analysis Workshop 16; duplicated and contaminated samples had been removed previously. Using the computer program PLINK [7], subjects were filtered who had less than 90% genotyping, and single-nucleotide polymorphisms (SNPs) were filtered that had less than 90% genotyping, Hardy-Weinberg equilibrium in controls *p *< 0.0001, or minor allele frequency (MAF) < 0.05. Using the computer program EIGENSTRAT [8], population outliers were removed who were >6 standard deviations from any of the first five principal components (PCs) identified in PC analysis.

First we analyzed the whole-genome data using structured association analyses of EIGENSTRAT; although the NARAC cases and controls are Caucasian, differences in intra-European ancestry [9] can produce false-positive associations. SNPs used for the PCA were filtered to remove regions of extended local associations (chr 8: 8-12 Mb, chr 6: 24-36 Mb, chr 11: 42-58 Mb, chr 5: 44-51.5 Mb, and chr 17: 40-43 Mb) and pruned to have *r*^2 ^< 0.2 within a sliding window of 1 kb with a step size of 100, similar to methods of Fellay et al. [10] and Hom et al. [11]. We included correction for the first six PCs (see Results).

We used a conservative genome-wide significance threshold of *p *= 1 × 10^-8^. Because all SNPs exceeding this threshold (i.e., lower *p*-value) were in the MHC in a region of extended linkage disequilibrium (LD), we proceeded with conditional analyses to attempt to establish signals that were independent of the shared epitope and each other. We modeled the SE as a multi-allelic marker with values corresponding to negative, low-risk, or high-risk. SE alleles were considered high risk if they were one of DRB1*0401, 0404, 0405, 0408, or 0409. Table 1 shows the case-control ratios for each risk category.

**Table 1:** *HLA-DRB1 *risk levels. Definitions and case-control ratios for shared-epitope (SE) categories.

DRB1 risk level	Definition	Case-control ratio
3 = Highest-risk SE	DRB1*0401, 0404, 0405, 0408, or 0409	72%-28%
2 = Lower-risk SE	Other SE alleles	52%-48%
1 = No SE	Not an SE allele	23%-77%

Our conditional analyses, using the computer program Whap [12], proceeded as follows. Starting with the SE as the top marker, we tested each two-SNP marker (SE plus each other SNP) for independence of the other SNP; in particular this uses a likelihood-ratio test to determine the significance of the difference between the two-SNP "alternate" model versus the one-SNP (SE in this case) "null" model. As long as the most significant SNP was <0.001, we added this best SNP to the list of independent SNPs, and proceeded to test all three-marker combinations compared to our best two-SNP model; and so on with larger haplotypes. In the final list, we also tested each locus for a significant addition to the model containing all other top SNPs.

## Results

After quality control filtering (above), 486,078 SNPs remained for the whole-genome analyses. All subjects had genotyping >90%, and eight controls were removed as outliers detected by EIGENSTRAT, leaving 868 cases and 1186 controls for the final analyses. In structured association analysis with EIGENSTRAT, we corrected for the first six PCs because the scree graph of eigenvalues levelled off at the sixth component. PCs one, two, four, and five were all highly significant in association tests with cases and controls (all *p *≤ 10^-8^). In this whole-genome analysis we identified exactly 200 SNPs with *p *< 10^-8^, all between 30.38 Mb and 33.08 Mb in the MHC region. Table 2 shows the significance of associations in this dataset for known RA risk alleles outside of the MHC [2,3,13-17]; for SNPs not in the Illumina 550k panel, there were perfect proxy SNPs (*r*^2 ^= 1) in the HapMap CEU population [18]. Although not reaching our 10^-8 ^threshold, we observed *p*-values from 10^-5 ^to 5 × 10^-6 ^for *PTPN22 *and *TRAF1-C5 *SNPs, and *p *= 0.03 for *STAT4*. This dataset was underpowered to detect any of these risk alleles at a genome-wide level for their published odds ratios (ORs); for example, we had approximately 70% power to detect the highest OR of 1.75 (*PTPN22*, MAF = 11%) at *p *= 10^-8^, and only 50% power to detect the lowest OR of 1.15 (*CD40*, MAF = 25%) at *p *= 0.05.

**Table 2:** Significance of associations with published RA risk alleles

SNP (GENE)	Reference	Proxy (*r*^2 ^= 1)	EIGENSTRAT adjusted *p*-value
rs2476601 (PTPN22)	[16]	N/A	5.3 × 10^-6^
rs7574865 (STAT4)	[2]	N/A	0.034
rs3761847 (TRAF1-C5)	[3]	N/A	1.1 × 10^-5^
rs1953126 (TRAF1-C5)	[17]	N/A	8.0 × 10^-6^
rs10499194 (TNFAIP3)	[18]	rs13192841	0.25
rs6920220 (TNFAIP3)	[19]	rs6933404	0.17
rs4810485 (CD40)	[20]	rs1569723	0.12

**Figure 1:**
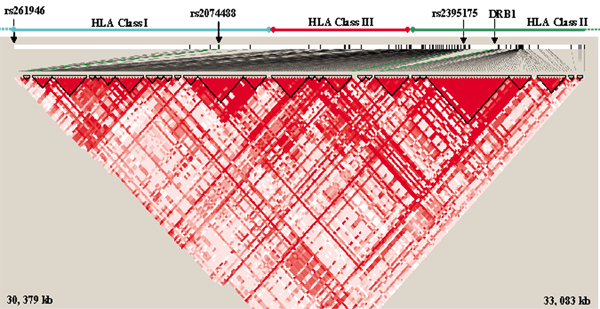
**Region with LD of SNPs studied in conditional analyses**. Haploview diagram displays increasing red with higher D'. Three independently significant RA SNPs and *HLA-DRB1 *locations are indicated.

Results of our conditional analyses are shown in Table 3. Loci are shown in the order added by the algorithm (see Methods), i.e., rs261946 has the lowest *p*-value conditional on the SE, rs2074488 has the lowest *p*-value conditional on both SE and rs261946, and so on. Out of the 200 SNPs, three independent signals were evident in addition to the SE risk levels. One signal is located in the classical HLA class II region between genes *BTNL2 *and *HLA-DRA*, and two signals are in the classical HLA class I region (see Figure 1) near *TRIM39 *and *HLA-C*.

**Table 3:** Independent SNPs in conditional analysis of RA susceptibility

Locus (sequence #)^a^	Unadjusted single-marker *p*-value from haploview^b^	Single-marker EIGENSTRAT *p*-value (rank)	*p*-value conditional on loci above^c^	*p*-value conditional on other 3 loci^c^	Location (kb)	Closest gene(s)
Shared epitope (#129)	1.9 × 10^-188^	N/A	N/A	8.6 × 10^-74^	32,655-32,666	In DRB1
rs261946 (#1)	7.2 × 10^-17^	1.9 × 10^-9 ^(185)	0.00019	0.0027	30,379	TRIM39
rs2074488 (#5)	2.3 × 10^-24^	5.0 × 10^-12 ^(151)	6.6 × 10^-5^	0.0011	31,348	HLA-C
rs2395175 (#119)	1.4 × 10^-117^	1.9 × 10^-80 ^(2)	0.00031	0.00031	32,513	Between BTNL2 and HLA-DRA

^a^# indicates marker position in Figure 1. Loci are in order added by conditional algorithm (see Methods).

^b^For shared epitope, multi-allelic SNP test in Whap using No-Low-High risk.

^c^Whap log ratio test using all loci as alternate model versus null model without this locus.

Table 4 shows the case-control frequencies and ORs for the final haplotypes, with the most common haplotype (ACG-1) as the reference haplotype. The highest-risk haplotype of these SNPs and the SE level had OR = 27.2 (95% CI, 16.7-44.4) in comparison to OR = 7.3 (95% CI, 4.7-11.1) overall comparing the SE risk levels alone (data not shown).

**Table 4:** Frequencies and odds ratios for haplotypes of three SNPs and shared epitope proxy risk level

Haplotype	Frequency in cases	Frequency in controls	OR (95% CI)
GAA-3	9.80%	1.90%	27.2 (16.7-44.4)
AAA-3	6.50%	1.50%	22.2 (12.6-39)
ACA-3	17.10%	4.40%	19.9 (13.4-29.7)
GCG-2	5.30%	2.40%	14.6 (8.6-24.8)
GCA-3	12.60%	4.20%	13.1 (9-19.1)
ACG-3	3.40%	1.80%	6.2 (3.7-10.4)
ACG-2	13.80%	9.50%	5.2 (3.8-7.1)
AAG-1	1.80%	2.70%	2.5 (1.4-4.5)
GAG-1	1.40%	2.40%	NS
GCG-1	6.00%	13.00%	NS
ACA-1	1.50%	2.50%	NS
ACG-1	20.80%	53.80%	(Reference group)

## Conclusion

In our analysis of the Genetic Analysis Workshop 16 dataset, there was insufficient power to detect known associations with RA susceptibility at a genome-wide significance level outside of the MHC; the most significant association was *p *= 5.3 × 10^-6 ^for *PTPN22*. Clearly, the MHC is the most influential genetic region in RA susceptibility, but extensive LD makes isolating the precise loci difficult. We have used conditional analyses as a tool to investigate the presence of multiple RA risk factors in the MHC region in addition to the SE. Out of 200 candidate SNPs having unconditional *p*-values < 10^-8^, we have identified an additional HLA class II marker and two HLA class I markers which have significant associations with RA susceptibility that are not fully explained by LD with *HLA-DRB1*. A better understanding of these genetic influences can be helpful in elucidating the complex genetic components of RA.

Previous studies of MHC effects on RA susceptibility beyond the SE have identified additional independent signals but have been largely inconsistent, due at least in part to the difficulty of narrowing down regions of association in the presence of extended LD [1,13]. Multiple studies have implicated the TNF-lymphotoxin locus in class III [1], which were not significant in our conditional analysis. Other studies also using NARAC cases have observed signals in class I [5,14], including *HLA-C*, our second SNP added in conditional analysis. Our first SNP is in the gene *TRIM39*, also in class I but not previously implicated. Our third SNP, in class II, is 150 kb upstream from *HLA-DRB1 *between the *BTNL2 *and *HLA-DRA *genes. *BTNL2 *has been associated with RA, systemic lupus erythematosus, and type 1 diabetes [15]; this is attributed to its association with predisposing *HLA DQB1-DRB1 *haplotypes, which may explain its presence in our data as well.

It is important to note that the NARAC population is primarily Caucasian. Other populations could have quite different distributions of these haplotypes as well as other haplotypes and allele frequencies. A similar analysis in other ethnic groups could be very informative.

## List of abbreviations used

LD: Linkage disequilibrium; MAF: Minor allele frequency; MHC: Major histocompatibility complex; NARAC: North American Rheumatoid Arthritis Consortium; OR: Odds ratio; PCA: Principal components analysis; RA: Rheumatoid arthritis; SE: Shared epitope; SNP: Single-nucleotide polymorphism

## Competing interests

The authors declare that they have no competing interests.

## Authors' contributions

KET performed statistical analyses and drafted the manuscript. LAC recruited patients as part of the NARAC collaboration. LAC and KET designed the study, revised the manuscript, and read and approved the final manuscript.
